# Effect of Web atmospherics and satisfaction on purchase behavior: stimulus–organism–response model

**DOI:** 10.1186/s43093-021-00107-3

**Published:** 2021-11-08

**Authors:** Abbas N. Albarq

**Affiliations:** School of Economics and Administrative Science, Al Imam Muhammad ibn Saud Islamic University, Riyadh, 11431 Saudi Arabia

**Keywords:** Online shopping, Web satisfaction, Structural equation modelling, Website cues

## Abstract

The effect of Web atmospheric clues on the purchase intention of Jordanian shoppers has been evaluated in this study, along with the interventions of Website gratification. The primary data collection for testing the research model via a survey method has been performed from Jordan's capital city, namely Amman. The tenure of data collection from Amman is from July to January 2020. The Structural Equation Modeling method was used to analyze the data with AMOS 22.0 software. Convergent and discriminatory legitimacy of the measurement model has been estimated with the use of confirmatory factor analysis. The satisfaction component effectively negotiates the impact of Website clues on the intent of purchase. Moreover, the purchase intention is a consequent effect of the satisfaction caused by these preliminary factors. The e-retailers and marketers of Jordan are able to interpret the influence of multiple stimulating factors on the satisfaction gained from Web-related services with the help of the outcomes of this study. It is the prerogative of online retailers to ensure the delivery of the strongest atmospheric clues impacting the Website satisfaction to the shoppers. In the context of Jordanians, this study establishes that Web managers should designate a higher number of resources to the clues that enhance the excitement value of the atmospherics of Web portals. This study boosts the knowledge of the researchers having academic interest and practical inclination toward the aspects of developing economies and adds to their current level of knowledge regarding e-retailing and online buying behavior.

## Introduction

Over the years, technology has revolutionized our world and daily lives, e-commerce tends to attract numerous online buyers who utilize a variety of devices to do online shopping. As a result of the surging trends of online shopping and globalization, the global online business environment is undergoing evolution to satiate the growing demands of online purchasing. The social distancing norms enforced due to the current coronavirus pandemic across the globe will force the customers to quit shopping from brick and mortar stores, thereby leading to an exponential growth of e-commerce.

The raging growth of e-commerce sales and cutthroat competition arising due to it have forced many entrepreneurs to engage Web atmospherics as a strategic approach of differentiation, where the Web atmospherics can be defined as "any Web interface component within an individual’s perceptual field that stimulates one’s senses" [31, p. 230], to create positive effects (e.g., positive affect, positive cognitions, etc.) to increase favorable consumer responses (e.g., site revisiting, browsing, etc.). When marketers design Web interfaces in order to entice consumers, they are utilizing Web atmospherics [29]. Web atmospherics influence affective and cognitive online shopping experience [30].

Under such strategies, theme-based websites are developed by entrepreneurs to stimulate the finest quality emotional and intellectual stature of online customers. Contemporary studies [29, 34] tend to assess the behavior of consumers with special reference to the time and money intended to be consumed in online shopping. Additionally, entrepreneurs keenly outline the Web environment to upgrade the online shopping experiences as well as client communications of customers [14].

However, accessing online platforms via smartphones and broadband connectivity continues to be a challenge due to multiple constraints. Regardless of such immense growth, "online purchasing in Jordan faces many demanding situations, Jordanian clients are cash-oriented," as quoted by [6]. Challenges linked with e-trade and online shopping are still prevalent in Jordan [46]. Hasan and Morris [27] have identified multiple constraints experienced by the customers that are related to website satisfaction gained from Arab e-trade portals. These issues can arise due to unavailability of merchandise, complex ordering process, inefficiency to read small fonts, lack of alternative delivery options, ambiguity in website content, and erratic language (not consistent or regular). The efforts of multiple researchers to outline the limits of e-commerce in Jordan remain superficial; probably, due to the resource scarcity and inadequate interest of Jordanian consumers in e-commerce [6].

As stated by [17], an increase worth 4.8% has been witnessed in online purchasing trends. However, as opined by [2], a decline from 14% in 2011-2-12 to 8% in 2015–2016 has been seen in the online shopping trends. Ministry of Information and Communications Technology [37] pointed out that irrespective of the plethora of e-commerce options available in Jordan, the sales between citizens and international organizations exceed the sales between citizens and local organizations in Jordan. The reason for the hostile attitude of Jordanian customers toward online shopping is unknown even after the increased prevalence of e-commerce in Jordan as compared to other eastern countries. Few causes like traditional beliefs, online safety and banking enterprises constraints have been reported by some researchers [3, 6, 46].

Multiple aspects related to affordability, security, cybercrimes, shortage of payment tools and delivery issues have been highlighted as limiting factors for e-trade in the former studies [4]. An insight into these aspects will enable the entrepreneurs to gain a better understanding of buyers' mindset and accordingly increase their investments in digital marketing that leads to enticing more customers. Hence, the objective/s of online buying was analyzed in [12, 13]. Additional matters linked with online website satisfaction [7, 8], online purchasing [5] and online shopping motivators [32] were contemplated and studied exhaustively. Equivalent research works have demonstrated that multiple values and extrinsic Web atmospherics, in addition to the demographic and socio-cultural factors, strongly impact the purchase behaviors and intentions of online customers. A noticeable gap has been witnessed in the literary studies associated with purchase behaviors and consumption trends in the Middle Eastern regions as a maximum number of former studies have been done on the USA and European nations [25]. Nonetheless, rarely any studies have been focused on researching the buying intention (net atmospheric elements) in Jordan or the online shopping and advertising patterns. Hence, online shopping models and trends have been excluded from this study and much focus has been laid on preliminary factors linked with online buying behavior and their importance in affecting the buying intent of Jordanian customers.

This research specifically intends to enrich the existing literary studies by manifesting the relevance of Web atmospherics and creating a manageable online environment that enhances Web satisfaction through the application of the stimulus–organism–response (S-O-R) approach, which elaborates the online shopping behaviors of customers in Jordan. In this study, the effect of atmospheric clues on the online buying behavior of Jordanian buyers (organisms) has been explained along with the intervention of Web satisfaction in Web atmospherics (stimuli) and resultant buying behavior (reaction). This study adds power to the existing literature studies by assessing the potential factors that impact the inclination of consumers toward online buying behavior by focusing on Web atmospherics and satisfaction.

## Literature review

### S-O-R Model

The S-O-R model is based on the theoretical grounds of environmental psychology suggested by [36]. According to this framework, stimuli can be defined as preliminary criteria that impact the emotional conditions of consumers (organisms), thus altering the consumers' behaviors and intent to purchase. The retail industry has been the pioneer to implement the S-O-R model firstly ever [19], under which the stimuli function as environmental signals (atmospheric clues) due to which the organisms tend to react in a distinctive manner of acceptance or avoidance, owing to their cognitive and emotional stature. Relevant tests have been done on traditional retail stores to assess the effectiveness of the S-O-R model through multiple studies [41, 43]. In the S-O-R model, the implication of stimuli corresponds to the specific characteristics affecting the perceptions of consumers [35]. These characteristics mark the onset of particular consumer behavior, whereby these cues impact the customers' cognition by giving a direct or indirect kick toward a specific behavior [43]. The characteristics acting as stimuli in the retail sector encompass social attributes like customers and employees in the stores, designing of space to create certain buyer effects, specifically, the designing of buying environments to produce specific emotional effects in the buyer that enhance purchase probability.

The intervention felt by the organisms internally between these stimuli forces them to react in a specific manner. Under this cycle, the stimuli are transformed by the customers into significant insights, thereby inferring that notions and perceptions regarding special tasks can illustrate the individual's feelings and psychological state [36]. The reaction implies the counteraction of a person, which includes both attitudinal as well as behavioral responses. The implementation of the S-O-R model in a study revealed the extensive bonding between aesthetics of Web, supplier quality, and contentment [44]. Additionally, the interconnection between pleasure and stimulation provoked by Web atmospherics and customers' purchase objectives, dignity and buying approach has been revealed by delving into the impact of website cues on online shoppers [24]. The usage of the S-O-R model has been recommended by this study via integration of shopping principles (utilitarian and hedonic) as intrinsic motivators and Web environmental clues (pleasure, facts and efficacy) as extrinsic motivators. Herein, Web satisfaction (WS) corresponds to the organism, whereas customers' purchase objectives imply behavioral response (R).

### Web atmospheric

In the case of a typical retail shop, atmospherics can be defined as the "conscious design of the distance to create unique outcomes on shoppers" [31, p. 231]. The atmospherics and milieu of the retail stores act as crucial elements in comparison to advertising investments [18] and can even be implemented in online retailing. As per a proposition, digital traders build a Web environment that assists in impacting consumers' viewpoint regarding the Web as well as improving their buying experiences [28].

With the expansion of the notion of atmospherics to the context of online purchasing, Web atmospherics is reviewed as "a careful designing of net environments to create conducive effects among customers so that it will increase favorable responses" [14, p. 482]. Prashar et al. [41] highlighted the similarity between the atmospherics of online stores and conventional brick and mortar stores as, in both cases, it facilitates significant information linked with the store that influences the behaviors and outcomes of customers. Hence, online customers are inspired to develop their Web ecosystem in order to impact their perception regarding Web stores and improvise their purchasing experiences [41]. The studies of [15, 21] assess the environmental characteristics of the Web platforms, including the color, style and size of the font and music [15, 21]. As opined by the authors, the preliminary environmental clues strongly impact the customers' emotions (pleasure and stimulation), wherein the effect is digitalized and paralyzed. The traits of a website such as cognizance and an efficiency have been explored and examined in additional studies [18, 28, 31, 41].

It is an inherent ability of online customers to memorize the Web portals' display which assists them in repetitive and easy shopping of products and services by preserving time. Facilitation of accurate, relevant and innovative data by the websites reduces and the time and energy consumption of customers used for information hunting, as a result of which, impressive reviews are being earned [30]. A comprehensive model [34] discusses the emotional and cognitive elements of online buying behavior via an exploration of the connection between consumers' notion of Web atmospherics and their emotional stimulants like dignity, motivation and supremacy. Resultantly, consumers' approach toward the websites and their merchandise, website engagement and consumers' buying intent is largely affected by this notion. Web Entertainment (WE) and Web Informativeness (WI) are significant characteristics of websites due to which the Web is recognized as a source of "infotainment" [20]. Despite the significance of WI as a crucial function, the connotation of the category of data and the presentation of data cannot be ruled out. According to Prashar et al. [41], the efficacy of factual content material (EIC) indicates the completeness, relevance and novelty of the data offered by digital platforms from the customers' perspective.

Taking this into account, the below-mentioned hypotheses have been recommended:

#### **H**_**1**_

A positive and substantial connection exists between online shopping Website Informativeness (WI) and Website Satisfaction (WS).

#### **H**_**2**_

A positive and substantial connection exists between online shopping Website Entertainment (WE) and Website Satisfaction (WS).

#### **H**_**3**_

A positive and significant connection exists between online shopping Effectiveness of Information Content (EIC) and Website Satisfaction (WS).

### Website satisfaction and purchase intention based on atmospheric cues

Buyers’ satisfaction has been illustrated as “the assessment of products and services done by consumers in line with their desires and expectations” [38]. Contentment is a state of mind that is felt after undergoing a favorable experience [45]. Business enterprises aiming to lure customers need to establish smooth interaction with consumers and assess their needs as well as purchase intentions on a regular basis. Purposeful shopping can be defined as the powerful role of the efforts put in by customers to do definite purchasing via internet platforms [33]. Anderson and Srinivasan [7, p. 125] defined consumers’ pride as “the satisfaction gained by consumers with respect to their prior purchase experiences with a particular e-commerce brand or organization.”

**Fig. 1:**
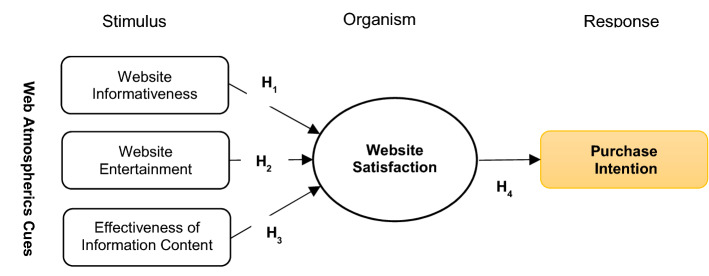
Proposed research framework

Multiple studies have attempted to outline the bond between the online shopping intention of consumers as well as the satisfaction gained by them through websites. Customers display a great extent of positive behavior after experiencing high-quality services from a website, which ultimately contributes to higher online purchase determination [41]. Likewise, it has been advocated that contentment experienced with a specific website results in a superior goal (purchase and repurchase) [18]. Albarq [5] stated that overall satisfaction received after shopping from online stores has a substantial influence on the intent to purchase from the same e-trader. Resultantly, consumers' purchase intentions linked with an internet service provider are driven by their behavioral propensity toward that website, which is a reaction to their ubiquitous pleasure gained from online shopping experiences [8]. Prashar et al. [41] secured the bonding between satisfaction realized by consumers from a website and their purchase intention (Fig. 1). Hence, in light of the fact that WS augments the chances of purchases on a website, the below-mentioned hypothesis has been proposed:

#### **H**_**4**_

A positive and important relationship exists between Website Satisfaction (WS) and the buyer’s Purchase Intention (PI) regarding a Web portal.

## Methods

### Measures

The variables substantiated in previous empirical studies have been used for the survey method adopted in this study. The number of tools used to gauge each variable and its source has been displayed in Table 1, in addition to the recent studies for testing these scales. The calibration of the items has been done through a seven-point Likert scale ranging from 1 to 7, wherein 1 implies strongly disagree, and 7 means strongly agree.

**Table 1:** Variables, number of items, and latest validation sources

Variable	No. of items	Items source	Recent validation
Effectiveness of information (EIC)	5	[10]	[1]; [41]
Website entertainment (WE)	5	[39]	[11]; [41]
Website informativeness (WI)	4	[41]	[41]
Website satisfaction (WS)	3	[10]	[1]; [41]
Purchase intention (PI)	4	[18]	[1]; [44]

The questionnaire was rewritten from English to the Arabic language by an Arabian professor. After identification, the enormity values of every structure were submitted to a control panel comprising of three members of Jordanian universities. Additionally, these dimensions were shared with marketing consultants too so that the questionnaire's validity could be ensured and then, suggested changes were incorporated in the questionnaire in line with the norms. The tools were tested on 42 respondents, and then it was finally booked for the survey after few alterations.

### Data collection

Jordan's capital city, Amman, was chosen for primary data collection between July to September 2019. The sampling has been done through the convenience sampling approach due to its convenience as well as the presence of data dispersed among survey respondents who were experienced in online purchasing, specifically the ones who had transacted online three or more times. The respondents were asked to confirm their online shopping transactions post, which their permission for participation was asked for. A total sample of 388 respondents was selected for the study, out of which 316 (81.4%) were found to be relevant for this study.

### Analysis

A combination of the Structural Equation Modeling (SEM) approach with AMOS 22.0 software and a dual-step procedure has been used to analyze the data in this study [26]. The convergent and divergent validities have been estimated by engaging the technique of confirmatory production unit analysis. The structural model framework and assumptions assisted in scrutinizing the tests.

## Results

The demographics of respondents have been illustrated in Table 2, which shows that 41.7% of respondents are men (*n* = 132) and 58.3% of respondents are women (*n* = 184). Most of the respondents are from the age category of 22–39 years (62.3%, *n* = 197), and most are married (57.3%, *n* = 181). The demographic data pertaining to monthly allowance or profitability was collated to determine their expenses in online shopping. The earnings of most respondents (86.4%) was above 1500 Jordanian Dinar (JOD), Jordanian Dinar exchange rate to USD (0.708).

**Table 2:** Demographic representation of respondents

Demographic group	Demographic category	Count	Percentage
Gender	Male	132	41.7
	Female	184	58.3
Age	Below 22 years	15	4.7
	22–39 years	197	62.3
	40–59 years	104	33.0
Marital status	Married	181	57.3
	Single	96	30.4
	Other	39	12.3
Education level	Students	0	0.0
	Graduates	203	72.9
	Postgraduates and above	113	27.1
Monthly income	Less than 600 JOD	0	0.0
	600 to 1499 JOD	43	13.6
	1500 to 2499 JOD	175	55.4
	More than 2500 JOD	98	31.0

The sufficiency of the framework measurements has been ascertained along with the overall interpretation. The recommended version seems to be sufficiently competent, as pointed out by the goodness of match inputs. The ratio of the least chi-square to the degrees of freedom (CMIN/df) was derived as 2.265 (Table 3), affirming a healthy correlation between speculated version and the database. The observations assisted in deriving values of GFI (0.894), adjusted GFI (0.906), CFI (0.923), IFI (0.928), TLI (0.917), Normed Fit Index (NFI) (0.91), and root-mean-square error of approximation (RMSEA) (0.069). The mathematical importance of the parameter projections that were examined was grounded on the *t* statistic (crucial ratio). High values mark the converging effectiveness (Table 3). The regulated loadings, the dimension framework and the *t*-values critical ratio have also been depicted in Table.

**Table 3:** Measurement prototype (CFA)

Factor and items	Factor loading	CR	*α*	AVE	Construct reliability
*Effectiveness of information content*			0.84	0.69	0.91
EIC-1	0.839	12.972			
EIC-2	0.863	14.261			
EIC-3	0.830	Deleted			
EIC-4	0.859	13.760			
EIC-5	0.802	Fixed			
*Web entertainment*			0.81	0.68	0.89
WE-1	0.797	10.477			
WE-2	0.847	Deleted			
WE-3	0.892	13.458			
WE-4	0.880	11.846			
WE-5	0.758	Fixed			
*Web informativeness*			0.84	0.61	0.85
WI-1	0.834	13.384			
WI-2	0.736	11.631			
WI-3	0.747	11.841			
WI-4	0.755	Fixed			
*Web satisfaction*			0.68	0.58	0.78
WS-1	0.768	9.894			
WS-2	0.798	11.188			
WS-3	0.698	Fixed			
*Purchase intention*			0.90	0.71	0.91
PI-1	0.801	14.894			
PI-2	0.885	16.112			
PI-3	0.901	17.152			
PI-4	0.798	Fixed			

Goodness of fit ratio: CMIN/df = 2.265; CFI = 0.923; GFI = 0.894; AGFI = 0.906; NFI = 0.91; IFI = 0.928; TLI = 0.917; RMSEA = 0.069

Revealed that the first estimate tries to identify the existence of a high interrelation between the projections of a particular structure or with that of other structures [23]. As shown in Table 4, the absolute value's (AVE) square root regarding one prototype is higher than the absolute value linked with the regulated correlation of the said structure with another structure involved in the analysis [AVE > correlation^2^].

**Table 4:** Discriminant validity for the measurement prototype

Construct	EIC	WE	WI	WS	PI
Effectiveness of information content (EIC)	0.732*				
Web entertainment (WE)	0.487	0.719*			
Web informativeness (WI)	0.651	0.632	0.721*		
Web satisfaction (WS)	0.648	0.681	0.701	0.832*	
Purchase intention (PI)	0.599	0.648	0.655	0.644	0.741*

^*^Implies the squared root of the AVE; others are correlation coefficients

Table 5 represents the comprehensive measurement framework, including the three extrinsically underlying structures (WI, WE, and EIC), the intrinsically underlying structure (WS) and reliant structure (purchase intention) that has been assessed to authenticate the recommended model and the assumptions. SEM performs the evaluation of the interrelationship existing between the structures. The structural framework projections have been represented in Table 4, which elaborates the vector effect of the standard path coefficients. The outcomes confirm a healthy fit of the recommended model with the data (CMIN/df = 3.38; CFI = 0.976; GFI = 0.967; AGFI = 0.903; NFI = 0.965; IFI = 0.976; TLI = 0.942; RMSEA = 0.071). Ha and Stoel [25] indicated that the RMSEA value approaching close to the mark of 0.08 enables the standardized root-mean-square residual (SRMR) to be used as a cue of the model fit.

**Table 5:** Regression weight of the testing outcomes of hypothesized prototype

Hypothesis	Estimate	Critical ratio	Result
H_1_: Web informativeness (WI) → Web satisfaction (WS)	0.23	4.61***	Supported
H_2_: Web entertainment (WE) → Web satisfaction (WS)	0.28	7.02***	Supported
H_3_: Effectiveness of information content (EIC) → Web satisfaction (WS)	0.31	8.24***	Supported
H_4_: Web satisfaction (WS) → Purchase intention (PI)	0.92	17.93***	Supported

Goodness of fit ratio: CMIN/df = 3.38; CFI = 0.976; AGFI = 0.903; GFI = 0.967; IFI = 0.976; NFI = 0.965; TLI = 0.942; RMSEA = 0.07; SRMR = 0.04. ***P < 0.001 and **P < 0.01

The results indicate that H_1, 2, 3_ are acknowledged, whereas WS is affected by WE (*β* = 0.28, *t* = 7.02***), WI (*β* = 0.31, *t* = 8.24***) and EIC (*β* = 0.23, *t* = 4.61***). This justifies *R*^2^ = 69% of the entire variance in WS. Subsequent assumption H_4_ marks the link between WS and buying intention of consumers for a particular website. The hypothesis is backed by the outcomes (*β* = 0.92, *t* = 17. 93***), which points toward the impact of satisfaction gained from a website on the purchase intentions of consumers having *R*^2^ = 62% of the total variance. Additionally, the direct influence of the three structures WE, WI, and EIC on WS and their indirect impact on purchase intent have also been portrayed in the results. WS affects the purchase intent directly and not indirectly. The indirect influence of WI (*β* = 0.31), WE (*β* = 0.25) and EIC (*β* = 0.17) has been reflected in the outcomes for analyzing the WS attributes in the purchase intention of customers. The overall impact is 0.91, reflecting the significant mediation done by WS during the determination of purchase intention. Hence, WS is a holistic mediator of online purchase principles, Web atmospherics and the purchase intent of consumers.

## Discussion

The engagement of the S-O-R framework enables this study to assess the online buying behavior and statistically investigates the contemplated associations impacting the said behavior. This study particularly examined the influence of three indicators of Web atmospherics, namely WI, WE and EIC, on the customers' WS, along with assessing its influence on their online purchase intention. The implementation appropriateness of the S-O-R framework in the context of e-commerce has been verified in this study that advances the existing literature based on Jordanian e-trade.

The findings indicate the impact of WI, WE and EIC on WS (organism), as a result of which the purchase intention also gets impacted [9, 16, 22, 42]. Hence, it is explicit that the external stimuli lay an influence on the Jordanian customers, thereby affecting their buying behavior. In the context of Jordan, WS intervenes in the connection between Web atmospheric indicators and consumers' online purchasing intention.

The research study is enormously significant for illustrating many aspects like values of online purchasing, Web atmospheric signals, website layouts and intent to purchase, which are successfully drawing the attention of multiple researchers. This research enriches the literature based on the e-commerce industry and online buying behavior, which benefits the people who are keen to participate in online purchasing in a developing nation like Jordan. The prospect highlights the impact of the trio of preceding attributes (exogenous latent) upon the consumers' WS (intervening factor) and purchase intention (endogenous latent). The characteristic WS interferes with the effect of online purchase principles and website indicators on buying intention. Hence, the study rules out the immediate impact of Web atmospherics on the behavioral consequences implying purchase intention and generates a need for integrating an intermediary factor (WS) to boost the purchase intention of consumers. These inferences authenticate the implementation of the S-O-R model in speculating the reactions of resultant behavior of consumers. The findings conveyed by Abrar et al. [1], wherein the impact of Web atmospherics on WS have been assessed, have been validated by the outcomes of this research also. In alignment with the research outcomes of Park et al. [40], this research confirms the immediate effect of WS on the purchase intention (reaction) of consumers (organisms), thereby propounding the need for the creation of attractive websites by the online retailers to enhance consumers' purchase intention. Moreover, retail business managers should persuade the strongest effect of Web atmospheric clues on the WS gained by customers. The research in the context of the Jordanian market environment advocates an increased stake in digital marketing.

Leveraging the most effective principles like website design standards in WS transforms the website into a more attractive, artistic and fascinating platform. These principles augment the informativeness of the website, which necessitates for the managers to maintain easy-to-upload, relevant and appropriate data or content. It is advisable for online traders to create extraordinary layouts, including the overall organization, color patterns and clear designs. It is imperative for websites to offer convenient transaction methods for attracting and retaining customers.

## Conclusion

The current research construes upon the reason and manner in which ‘pride with website’ adds to the principles of purchasing and website atmospherics inherent to the behavioral consequences via demonstration of its intervening role. While aspiring to enhance the customers’ experiences of shopping, online retail business owners must attempt to generate exceptional website atmospherics. Any country that desires to improvise its e-commerce businesses must adopt the theoretical connotations derived from this study.

Additionally, the study fosters the theoretical articulation for explaining the online shopping behavior of Jordanians. With the extension of previous studies’ inferences on the interrelationship between intrinsic elements and online purchasing behavior [4], this study postulates the direct impact of S-O-R on consumers’ WS and its indirect effect on the online purchase intention of consumers. The findings of the research conducted by Mazaheri et al. [34] marked the impact of Web atmospheric clues on WS, which have been fortified through this study. In harmony with the outcomes of the study performed by Albarq [3], and Park et al. [40], the current research corroborates the impact of customers' WS on their purchase intention. Thus, it can be concluded that if the websites are able to induce a high satisfaction rate among the online buyers, only then would they be able to generate positive purchase intentions among the online customers off Jordan.

Even though the current study has evaluated multiple topics of e-commerce comprehensively, it bears certain limitations that need to be focused on in further studies. Since this research was undertaken on a limited sample of Jordan (Amman), it is assumed that the conclusions are prone to change in case of replication of this study on alternative economic environments, countries, or unique commercial instances. Moreover, the study lacks in focusing on the core context of purchasing. Prospective studies can examine the alterations in consumers' reactions by including variable purchase contexts.

## Data Availability

The data that support the findings of this study are available at would be provided upon request.

## Abbreviations

EIC: Effectiveness of information content
WE: Web entertainment
WS: Web satisfaction
PI: Purchase intention
S-O-R: Stimulus–organism–response
CFA: Confirmatory factor analysis
AVE: Average variance extracted
RMSEA: Root-mean-square error of approximation
CR: Critical ratio
SEM: Structural equation model
